# A Simple Microfluidic Chip Design for Fundamental Bioseparation

**DOI:** 10.1155/2014/175457

**Published:** 2014-01-08

**Authors:** Alan S. Chan, Michael K. Danquah, Dominic Agyei, Patrick G. Hartley, Yonggang Zhu

**Affiliations:** ^1^CSIRO Materials Science and Engineering, Highett, VIC 3190, Australia; ^2^Department of Chemical Engineering, Monash University, Clayton, VIC 3800, Australia; ^3^Department of Chemical Engineering, Curtin University of Technology, Sarawak 98009, Malaysia

## Abstract

A microchip pressure-driven liquid chromatographic system with a packed column has been designed and fabricated by using poly(dimethylsiloxane) (PDMS). The liquid chromatographic column was packed with mesoporous silica beads of Ia3d space group. Separation of dyes and biopolymers was carried out to verify the performance of the chip. A mixture of dyes (fluorescein and rhodamine B) and a biopolymer mixture (10 kDa Dextran and 66 kDa BSA) were separated and the fluorescence technique was employed to detect the movement of the molecules. Fluorescein molecule was a nonretained species and rhodamine B was attached onto silica surface when dye mixture in deionized water was injected into the microchannel. The retention times for dextran molecule and BSA molecule in biopolymer separation experiment were 45 s and 120 s, respectively. Retention factor was estimated to be 3.3 for dextran and 10.4 for BSA. The selectivity was 3.2 and resolution was 10.7. Good separation of dyes and biopolymers was achieved and the chip design was verified.

## 1. Introduction

High-performance liquid chromatography (HPLC) is a widely used separation technique with numerous implementations in both preparative and analytical systems [1–4]. A wide variety of chromatography media available provides different requirements for various molecular separation modes. The miniaturized HPLC system would offer the advantage of smaller sample size, reduction of dead volume, lower solvent consumption, faster, higher-throughput analysis, and portability of the analytical system, enabling on-site and remote analysis [5, 6]. Despite these advantages, miniaturization of chromatographic systems needs to address some technical issues such as fabrication of chip-based chromatographic systems without compromising separation efficiency [6]. One such challenge is the introduction of stationary phase materials into a microfabricated microchannel [7].

Numerous examples of chip-based chromatographic systems in pharmaceutical and biomedical applications have been reviewed extensively [6, 8–10].

Open-tubular liquid chromatography microchips integrated with a sample injector and electrode demonstrated low chromatographic efficiency [11]. The low efficiency could be attributed to small surface area and relatively large injection volume of the system. A microfabricated device with C18 coated channels was used to demonstrate on-chip phase extraction [12]. However, using a separation column packed with beads may yield better separation efficiency because of higher available surface area per unit volume and reduced diffusion distances through the narrow fluid paths between neighbouring particles [13].

Several microchips with porous polymer monoliths formed in channels via photoinitiated polymerization have been reported [14–18]. Reversed-phase silica particles are also widely used as the stationary phase in HPLC and solid-phase extraction for preconcentration and separation of analytes or to remove unwanted components from samples [19–24]. Monolithic silica prepared by sol-gel process has been used as a stationary phase in separation columns by several researchers [25–28]. Wolfe et al. [29] also reported that silica beads packing provides higher extraction efficiency than a silica network synthesized via sol-gel chemistry. The chip preparation technique generally reported in the literature requires a chemical/thermal process and etching on chip for pattern design. The deep reactive ion etching on quartz chips has made the technology too labor intensive and expensive [30].

A number of approaches for particles trapping inside a microchannel have been reported. These are based on magnetic susceptibility [31], flow profile [32], and chemical treatments [33]. However, the easiest integrated method is the use of mechanical barriers that hinder the flow of particles and this could be a dam (horizontal) or pillar (vertical) structure [34]. Dam structure is simple to fabricate, but it limits the flow of liquid dramatically and results in nonuniform flow profiles. Pillar-type bead filters allow uniform liquid flow with smaller flow resistance [35], but microfabrication of pillar-type bead reservoirs is very difficult compared to dam type. An additional step in the fabrication process is required to cater for the internal structure inside the channel. Also, in the microfabrication, high precision micromachining must be obtained.

In this paper, the fabrication of a cost-effective and easy structured multilayered pressure-driven microchip for reversed-phase liquid chromatography in poly(dimethylsiloxane) (PDMS) is presented. Poly(dimethylsiloxane) (PDMS) is the most dominant polymeric material for microfluidics. This is due to its unique properties such as elastomeric properties, biocompatibility, optical transparency down to 280 nm, hydrophobic surface chemistry, pliability and ease of molding into micron size, and low manufacturing costs [10]. Reversed-phase mesoporous silica was used as a stationary phase in the bottom layer of the microchip liquid chromatographic system. The silica was trapped in between two polyethylene membranes acting as porous frits. Injection chamber and separation channel were fabricated on the top layer of the chip. The performance of the microchip was demonstrated by the separation of the dye mixture, fluorescein and rhodamine B, and the biopolymer mixture of 10 kDa Dextran and 66 kDa bovine serum albumin (BSA).

## 2. Microchip Design and Fabrication

**Figure 1:**
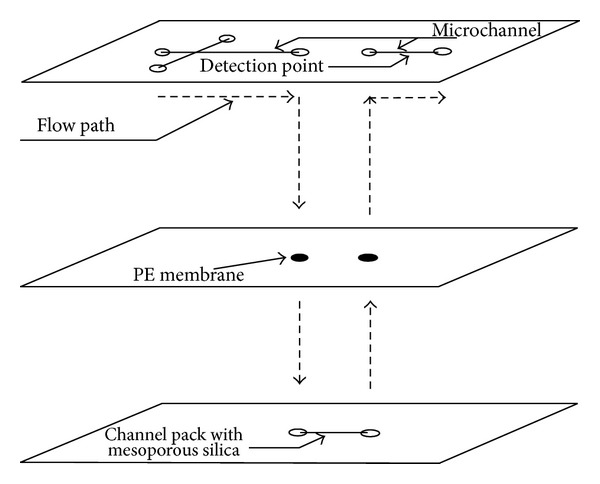
Schematic design of a poly(dimethylsiloxane) microfluidic chip with integrated HPLC column using mesoporous silica. The dimension of the channel cross-section is 70 *μ*m depth and 100 *μ*m width. The diameter of reservoirs and PE membrane port is 2 mm. The silica column dimension was 5 mm long, 70 *μ*m deep, and 100 *μ*m wide.

The proposed microfluidic chip design is aimed at improving the separation efficiency and resolution for macromolecules by integrating a HPLC column with mesoporous silica as the stationary phase. The designed chip consists of a mobile-phase channel, an injection channel, a packed silica column, and detection channel. The design consists of three layers of microstructures shown in Figure 1. Various microfluidic ports and separation capillary were constructed in the top layer of the PDMS substrate. Two polyethylene membranes were integrated into the second layer of the chip. These membranes serve as porous frits that keep the silica particles inside the HPLC column at the bottom layer and prevent it from leaking into the microchannels.

**Figure 2:**
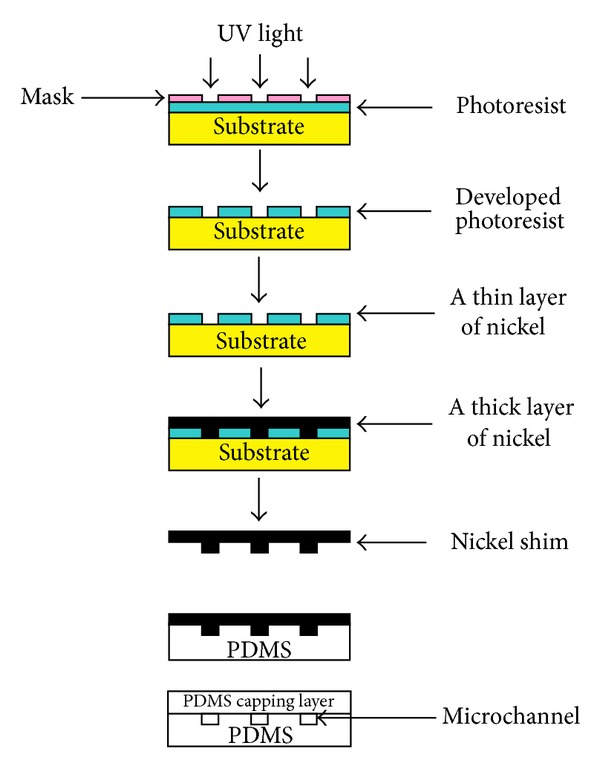
Schematic drawing of fabrication of nickel shim for microfluidic chip manufacturing.

A microfluidic chip was fabricated using a mold created by soft photolithographic technique shown in Figure 2. Microchip design patterns were drawn using commercial drawing software (Adobe Illustrator CS4) and printed directly onto a transparency film as a photomask. The photomask was then placed onto the multiple layers of dry film photoresist (Shipley 5038) that was laminated on a polished stainless steel. The microfluidic chip design was patterned lithographically onto the photoresist by UV illumination (*λ* = 350–450 nm) through photomask. The UV source was operated at 20 mJ/cm^2^. The exposed pattern was then developed in a 20% Na_2_CO_3_ solution and replicated as a nickel shim by a two-stage nickel deposition process. A thin nickel layer of 100 nm in thickness was first coated onto the patterned surface by sputter deposition. Secondly, a nickel layer of 150 *μ*m in thickness was electrodeposited in a nickel sulphamate bath. A mixture of PDMS prepolymer base and curing agent with a weight ratio of 10 to 1 was poured over the shim and then degassed under vacuum before curing at 60°C for 2 h. A PDMS substrate with the desired features was obtained and the thickness of each layer of PDMS substrate was kept at ~2 mm. The dimension of the channel cross-section is 70 *μ*m of depth and 100 *μ*m of width. The diameter of the reservoirs and PE membrane port is 2 mm. The silica column dimension is 5 mm long, 70 *μ*m deep, and 100 *μ*m. About 2 mm holes were punctured through the reservoirs in the top layer and PE membrane ports in the middle layer of the PDMS substrate. The middle and bottom layers of PDMS substrate were irreversibly bonded after pretreatment by oxygen plasma and curing at 60°C for 2 h. A 2 mm PE membrane with pore size of 10 *μ*m was placed on one end whilst silica particles were packed by pulling vacuum on top of the membrane. Another PE membrane was then put on the other end after the surface of the combined substrate was cleaned. Finally, the combined substrate was irreversibly bonded with the top layer of the substrate through pretreatment by oxygen plasma and curing at 60°C for 2 h. After chip fabrication was completed, the external tubing was put into the reservoirs and sealed with glue.

## 3. Experimental

### 3.1. Materials

Sylgard 184 PDMS prepolymer base and curing agent were purchased from Dow Corning. Mesoporous silica was supplied by CSIRO Material Science and Engineering Division. The properties of mesoporous silica were reported as follows: pore geometry—cubic (Ia3d space group); pore size—10.5 nm; porosity—0.945 cm^3^/g, and surface area—731 m^2^/g. Fluorescein sodium salt, rhodamine B, and methanol were purchased from Ajax Fine Chemicals. Dextran Alexa Fluor 568, 10 kDa and BSA Alexa Fluor 488, 66 kDa were purchased from Invitrogen. Deionized water was prepared using Milli-Q system from Millipore. All reagents were of analytical grade.

### 3.2. Experimental Setup

**Figure 3:**
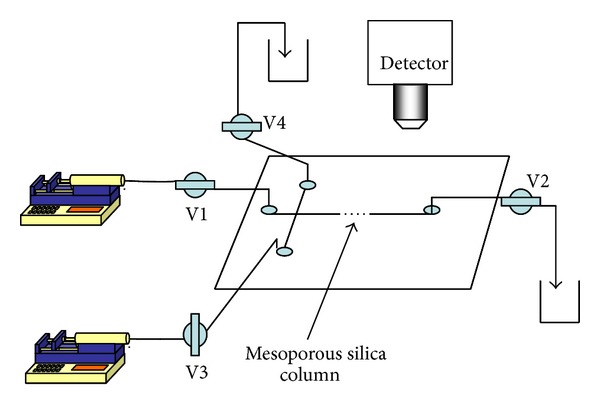
A schematic diagram of the chip LC system consisting of syringe pumps and inlet and outlet valves. Mobile-phase and sample injection into the microchannel can be achieved by operating injection pumps and valves in different sequences. Fluorescence technique is used to detect molecules at the separation channel.

In the present work, a dye mixture (fluorescein and rhodamine B) and a biopolymer mixture (10 kDa dextran and 66 kDa BSA) were used to demonstrate the applicability of a chip-based reversed-phase liquid chromatographic separation.  Figure 3 shows a schematic diagram of the chip LC system. The ends of the microchannels in the microchip were connected to four two-way stop cork valves (V1, V2, V3 and V4) via a separate plastic tube. The valves V1 and V3 were connected to a syringe pump and sample injection syringe pump, respectively. The chip LC system was placed on an inverted microscope with appropriate lens and UV band pass filters. Fluorescence technique with UV illumination was used to detect molecules. The incoming UV light beam is focused onto the separation channel. Emitted light from sample molecules is then directed to the photomultiplier (PMT) tube for experimental monitoring.

### 3.3. Methodologies

#### 3.3.1. On-Chip Injection

The mobile-phase inlet and outlet valves, V1 and V2, were opened while the sample inlet and outlet valves, V3 and V4, remained closed. The separation channel was flushed with the mobile-phase (DI water) until the baseline of the PMT tube output was stabilized. The sample plug was then introduced into the separation channel after the mobile-phase inlet and outlet valves (V1 and V2) were closed. Once the mobile-phase flow was halted, the sample inlet and outlet valves (V3 and V4) were then opened to load the sample using a syringe connected to the sample inlet valve V3 for 5 s after which the sample inlet and outlet valves were closed. The mobile-phase valves were then opened and the syringe pump for the mobile-phase was started.

For dye separation experiments, the sample containing 0.05 g/mL of fluorescein and 0.05 g/mL rhodamine B in deionized water was injected into the separation channel after the channel had been flushed with the mobile-phase. Deionised water was replaced by methanol as mobile-phase to elute the molecule attached onto the silica particles.

For biopolymer separation experiments, the sample mixture containing 0.25 mg BSA and 0.03 mg dextran in 1 mL of 0.01 M phosphate buffer saline (PBS) was prepared. The sample was loaded onto the silica column and the microchip was flushed with 0.01 M PBS buffer for 30 s to remove the remaining sample. The buffer was then replaced by a mobile-phase composition of methanol to elute the bounded biopolymers from the silica column.

## 4. Results and Discussion

### 4.1. Dye Separation

**Figure 4:**
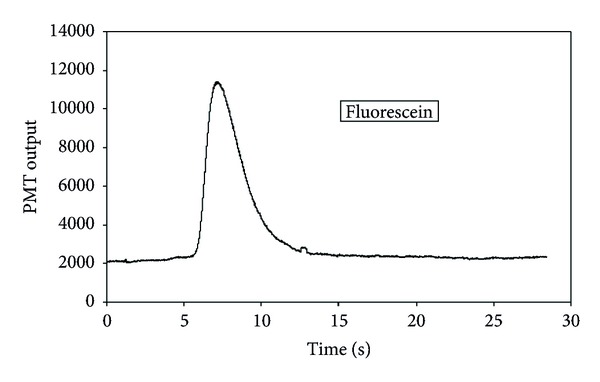
A chromatograph showing the single peak (fluorescein) observed right after the dyes mixture was injected into a separation channel at the flow rate of 0.23 *μ*L/s.

**Figure 5:**
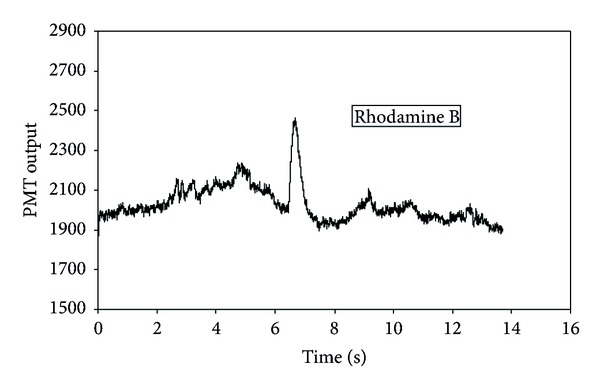
A chromatograph showing the peak for retained dyes observed during elution with methanol at a flow rate of 0.23 *μ*L/s.

**Figure 6:**
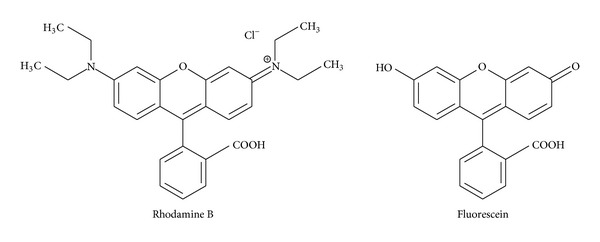
Chemical structures of rhodamine B and fluorescein.

After the mixture of dyes in deionised water was injected into the separation channel, only one peak was detected as shown in Figure 4. Methanol was then used to elute the retained dye from the silica, and this resulted in a single peak as shown in Figure 5. Microscopic analysis performed in between sample injection and elution showed that the silica particles turned red corresponding to the emission spectrum for rhodamine B. Thus, fluorescein molecules passed through the channel while rhodamine B molecules were retained in the silica packing. Hence, dyes were separated completely. The separation results, as expected, correspond to a conventional reversed-phase chromatography mechanism in which neutral molecules are separated in solution on the basis of their hydrophobicity [36, 37]. Solute retention increases with increasing polarity of the mobile-phase. A polar solvent, such as water, interacts preferentially with basic solutes such as amines. A comparison of the chemical structures rhodamine B (C_28_H_31_ClN_2_O_3_) and fluorescein (C_20_H_12_O_5_) is shown in (Figure 6). rhodamine B is therefore more likely to be retained on the surface of silica compared to fluorescein molecules.

### 4.2. Biomolecule Separation

**Figure 7:**
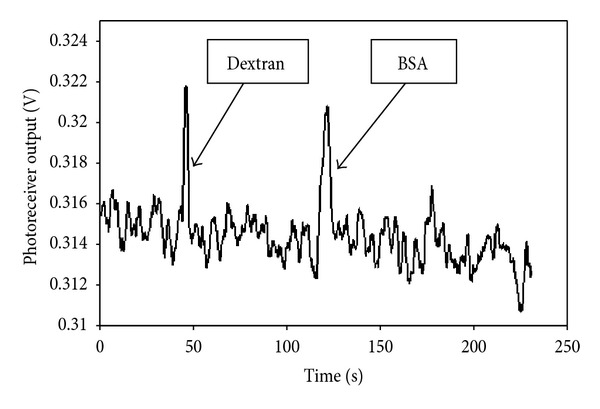
Biomolecule elution profile from the silica column with methanol mobile-phase at a flow rate of 0.23 *μ*L/s. Dextran was eluted prior to BSA.

Reversed-phase mode HPLC was performed on a chamber packed with mesoporous silica equilibrated with the PBS buffer. The biomolecule mixture sample was loaded onto the silica column. Both molecules were retained and Figure 7 shows the elution of the 10 kDa dextran and 66 kDa BSA, with methanol as mobile-phase at a flow rate of 0.23 *μ*L/s. The molecules were eluted and separated in 2 min. Under this condition, dextran was eluted prior to BSA. The dextran molecule used in this experiment is an anionic molecule and BSA is more hydrophobic. BSA is hydrophobic [38] and therefore will have a higher affinity for hydrophobic interaction with reversed-phase silica; hence, BSA was eluted later. Dextran is a neutral polysaccharide and as such will interact with PBS running buffer and elute first. The retention times for dextran and BSA molecules in the separation experiment were evaluated to be 45 s and 120 s, respectively. The retention factor was estimated to be 3.3 for dextran and 10.4 for BSA; hence, the selectivity (ratio of retention factor) was 3.2. Resolution (Rs), which describes the degree of separation between neighboring solute peaks, was calculated to be 10.7. Since the experimental value of Rs is higher than 1.5, the peak purity is 100%.

## 5. Conclusions

A pressure-driven liquid chromatography microfluidic device integrated with mesoporous silica as HPLC stationary phase on PDMS chip has been developed. This LC chip system illustratively provided favorable chromatographic separation for a dye mixture of fluorescein/Rhodamine B and biomolecule mixture of 10 kDa dextran and 66 kDa BSA. Hence, applications previously developed for a conventional HPLC system can potentially be adapted to the microchip LC system. The design of this microfluidic device can also be adapted for various biomolecular separation systems, such as capillary electrochromatography. This becomes feasible by choosing a suitable stationary and mobile-phase and can find use in applications such as point-of-care diagnostics and residue or pathogen detection in food and water.
